# Brain connectivity dynamics in cisgender and transmen people with gender incongruence before gender affirmative hormone treatment

**DOI:** 10.1038/s41598-021-00508-y

**Published:** 2021-10-26

**Authors:** Carme Uribe, Carme Junque, Esther Gómez-Gil, María Díez-Cirarda, Antonio Guillamon

**Affiliations:** 1 Medical Psychology Unit, Department of Medicine, Institute of Neuroscience, University of Barcelona, 08036 Barcelona, Spain; 2 Research Imaging Centre, Campbell Family Mental Health Research Institute, Centre for Addiction and Mental Health (CAMH), University of Toronto, Toronto, M5T 1R8 Canada; 3 Institute of Biomedical Research August Pi i Sunyer (IDIBAPS), 08036 Barcelona, Spain; 4 Centro de Investigación Biomédica en Red sobre Enfermedades Neurodegenerativas (CIBERNED: CB06/05/0018-ISCIII), 28031 Madrid, Spain; 5 Gender Unit, Hospital Clinic, 08036 Barcelona, Spain; 6 Neurodegenerative Diseases Group, Biocruces Bizkaia Health Research Institute, 48903 Barakaldo, Spain; 7 Departamento de Psicobiologia, Facultad de Psicologia, Universidad Nacional de Educacion a Distancia, 28040 Madrid, Spain

**Keywords:** Human behaviour, Neuroscience, Sexual behaviour, Social neuroscience

## Abstract

Large-scale brain network interactions have been described between *trans-* and *cis*-gender binary identities. However, a temporal perspective of the brain's spontaneous fluctuations is missing. We investigated the functional connectivity dynamics in transmen with gender incongruence and its relationship with interoceptive awareness. We describe four states in *native* and *meta-state* spaces: (i) one state highly prevalent with sparse overall connections; (ii) a second with strong couplings mainly involving components of the salience, default, and executive control networks. Two states with global sparse connectivity but positive couplings (iii) within the sensorimotor network, and (iv) between salience network regions. Transmen had more dynamical fluidity than cismen, while cismen presented less *meta-state* fluidity and range dynamism than transmen and ciswomen. A positive association between attention regulation and fluidity and *meta-state* range dynamism was found in transmen. There exist gender differences in the temporal brain dynamism, characterized by distinct interrelations of the salience network as catalyst interacting with other networks. We offer a functional explanation from the neurodevelopmental cortical hypothesis of a *gendered*-self.

## Introduction

The clinical neuroscience field has moved its interest from the classical studies of focal disconnections/increased connectivity in explaining pathology or cognition to the quantification of intrinsic functional brain organization^1,2^. More interestingly, the advent of integration/segregation models from systems neuroscience^3^ has enriched and expanded the study of brain function. In line with this framework, large-scale brain network interactions between four binary gender groups, namely transmen (TM) and transwomen with gender incongruence, cismen (CM), and ciswomen (CW) have been described^4^. It is important to note that while four groups were studied, participants identified themselves into two gender identities: either a man or a woman. The reported functional connectivity (FC) differences between groups were explained by the complementarity of the two dominant hypotheses in understanding the brain differences between gender groups: (i) a neurodevelopmental cortical hypothesis that suggests the existence of different brain phenotypes based on structural magnetic resonance imaging (MRI) data^5–8^ and genes polymorphisms of sex hormone receptors^9^; and (ii) a functional-based hypothesis explained by differences in regions involved in the own body perception^10,11^. The most compelling finding was the reduced FC in regions of the salience network and its couplings with the default mode, the sensorimotor, and the executive control networks when comparing the TM group with CM^4^.

However, these two predominant hypotheses do not explain the continuum of gender identity beyond the binary. A gendered-self is an inner self with maleness, femaleness, or other variants endowed. Gender identity may or may not correspond to a person’s sex assigned at birth^12,13^, and the classical dichotomy of *male* vs. *female* is being revisited^14^. Within the neuroimaging literature, nonbinary identities are yet to be studied, and the growing corpus of literature on gender minorities has focused on the gender incongruence of transgender binary people^10,11,15,16^. Such works help introduce the heterogeneity of the gender identity construct enriching the classical study of *female* and *male* differences in the brain. To date, FC studies investigating the sex/gender differences with large samples, do not account for well-characterized gender groups beyond the assumed self-identification to a cisgender identity^2,17–19^. In addition, the extensively reported differences between *male* and *female* brains over more than thirty years of functional neuroimaging research may have a probable reporting bias and excess of significance bias in the literature^18,20^.

To our knowledge, all studies that investigated the FC differences of transgender and cisgender individuals have employed a spatiotemporal stationary approach. This approach has brought plenty of tools based on the assumption that the statistical interdependence of time course between brain regions is constant across a whole resting-state acquisition^21^. However, a growing number of studies have described the brain as a complex system of rapid spatiotemporal transitions called brain dynamics^22–27^. This change of paradigm can significantly impact the study of gender differences, where we would not expect further than subtle brain structure and function changes, and network organization differences^17,28,29^. Following the characterization of stationary brain network interrelations between TM and CM groups^4^, we now aimed to investigate the functional brain dynamics from a sliding window approach from *native*^22^ and *meta* states^30^ spaces between TM, CM, and CW.

Furthermore, if we picture the cognitive function as a dynamic process supported by intrinsic structural and functional connectivity^21^, the study of the temporal brain transitions as underlying brain mechanisms to mentation^27,31^ and cognition^21,32^ is very promising. Thus, we were also interested in investigating the relationship of the spatiotemporal transitions with interoceptive awareness in TM. The functional hypothesis^11,33^ proposes a frontoparietal “*disconnection*” as a causal explanation of gender incongruence related to the own-body self-referential processing. This hypothesis is based on the Body Morph paradigm which accounts for body perception and gender identity^33,34^, while interoceptive awareness does not refer to the perception of the body but to the awareness directed towards the body and its sensations. Considering that we did find a decreased FC in the parietal and that key regions of the attentional network, i.e., the salience, the frontoparietal, and default mode networks were implicated in cismen and transmen differences^4^, we find this dimension worth exploring.

We hypothesized that we would find similar overall brain dynamic states among groups although specific dynamic metrics would differ between groups. Based on previous evidence^4,8^, TM and CW would differentiate from CM in the occurrence of states and/or in the brain intra and internetwork interactions within states. To address the study of interoceptive awareness, a self-report that measures the complexity of the construct from a multidimensional perspective was selected^35^. Although it is a complex and controversial concept, Mehling describes interoceptive awareness from propio and interoception channels understood as the processing of sensory inputs coming from the body^36^. We hypothesized that the brain dynamics states at rest would be related to interoceptive awareness in TM.

## Materials and methods

### Participants and instruments

Twenty-nine TM participants with no gender affirmative hormone treatment initiated (age:24.7 ± 6.2) were enrolled and diagnosed at the Gender Identity Unit of the Hospital Clinic of Barcelona. At the time of the recruitment, all TM met diagnostic criteria for gender identity disorder according to the DSM-IV-TR and ICD-10. However, to avoid stigmatization, the diagnosis was relabeled to gender incongruence according to the ICD-11 and as recommended by WPATH and EPATH^37^, while we followed the language guidelines proposed by Bouman et al.^37^.

Cisgender groups, namely 19 CM (age:22.2 ± 4.4) and 22 CW (age:19.6 ± 2.4), were enrolled from an advertisement in the Nursing Program at the University of Barcelona, and students were also asked to share with friends and acquaintances. Therefore, there were stark differences in the enrolment of transgender men and cisgender participants. To discount the presence of psychiatric disorders and substance abuse, the Mini-International Neuropsychiatric Interview was administered. In addition, cisgender participants were asked for acute or chronic medical conditions, and if they identified themselves as woman (CW), man (CM) or other non-binary identities^4^. Demographic information such as age and education can be found in the data article with the available images^38^.

Participants answered the Spanish version of the Multidimensional Assessment of Interoceptive Awareness (MAIA) questionnaire (available at https://osher.ucsf.edu/research/maia) divided in 8 dimensions, 32 items (from 0–5): *Noticing, Not-distracting, Not-worrying, Attention Regulation, Emotional Awareness, Self-regulation, Body Listening* and *Trusting*^35^.

Written informed consent was obtained from all participants after full explanation of procedures. The study was approved by the ethics committee of the Hospital Clinic of Barcelona, it was performed in accordance with relevant regulations and guidelines, and with consultancy from stakeholders of the transgender community.

### MRI acquisition and preprocessing

MRI were acquired with a 3 T scanner (MAGNETOM Trio, Siemens, Germany). Detailed protocol can be found elsewhere^38^. Briefly, T1 weighted images were acquired in the sagittal plane, TR = 2300 ms, TE = 2.98 ms, TI = 900 ms, 240 slices, FOV = 256 mm; matrix size = 256 × 256; 1 mm isotropic voxel. 240 T2* weighted images were acquired with a TR = 2500 ms s, TE = 28 ms, flip angle = 80º, slice thickness = 3 mm, FOV = 256 mm. Participants were instructed not to fall asleep and not to focus in any specific thought, keeping their eyes closed.

Basic preprocessing was conducted with AFNI using an in-house shell script, and included discarding the first five volumes, despiking with the 3DDESPIKE function, motion correction, grand-mean scaling, linear detrending (no temporal detrending) and temporal filtering using the 3D Fourier function with a frequency window of 0.1 Hz (low-pass) to 0.01 Hz (high-pass). Images were normalized into the standard MNI space and finally smoothed using a Gaussian kernel of 6 mm FWHM.

ICA-AROMA was applied for the automatic removal of motion-related artifact. See our data article^38^ for a summary of the groups’ means of all motion parameters. No motion parameter differed between groups.

### Stationary independent components maps

FC maps obtained from decomposing data using a group-level spatial independent component (IC) analysis were generated with GIFT v4.0. First, the number of IC for each participant was estimated using the Minimum Description Length criteria, resulting in a mean of 86 (SD = 77, range 9–192) components. Before data reduction, time series were *z-scored* to apply a voxel-wise variance normalization.

We then applied a: (i) *subject-specific data reduction* via principal component analysis (120 components); and (ii) *group data reduction* (100 IC) using the Expectation Maximization algorithm. Randomization was performed with the Infomax algorithm and repeated 20 times in ICASSO (https://research.ics.aalto.fi/ica/icasso/). Twenty-three IC with a stability index < 0.8 were excluded. Group IC were then back-projected with GICA^39^ and *z-scored*.

IC were spatially correlated^40^ and visually inspected. A total of 30 IC maps were finally selected. A 30 × 30 FC matrix was created with Fisher z-transformed scores of the peak-activity IC. The 30 IC underwent post-processing: timecourses were filtered using the 5th order Butterworth low-pass filter with a high frequency cut-off of 0.15 Hz, and temporally detrended.

### Functional connectivity dynamics analysis

**Figure 1:**
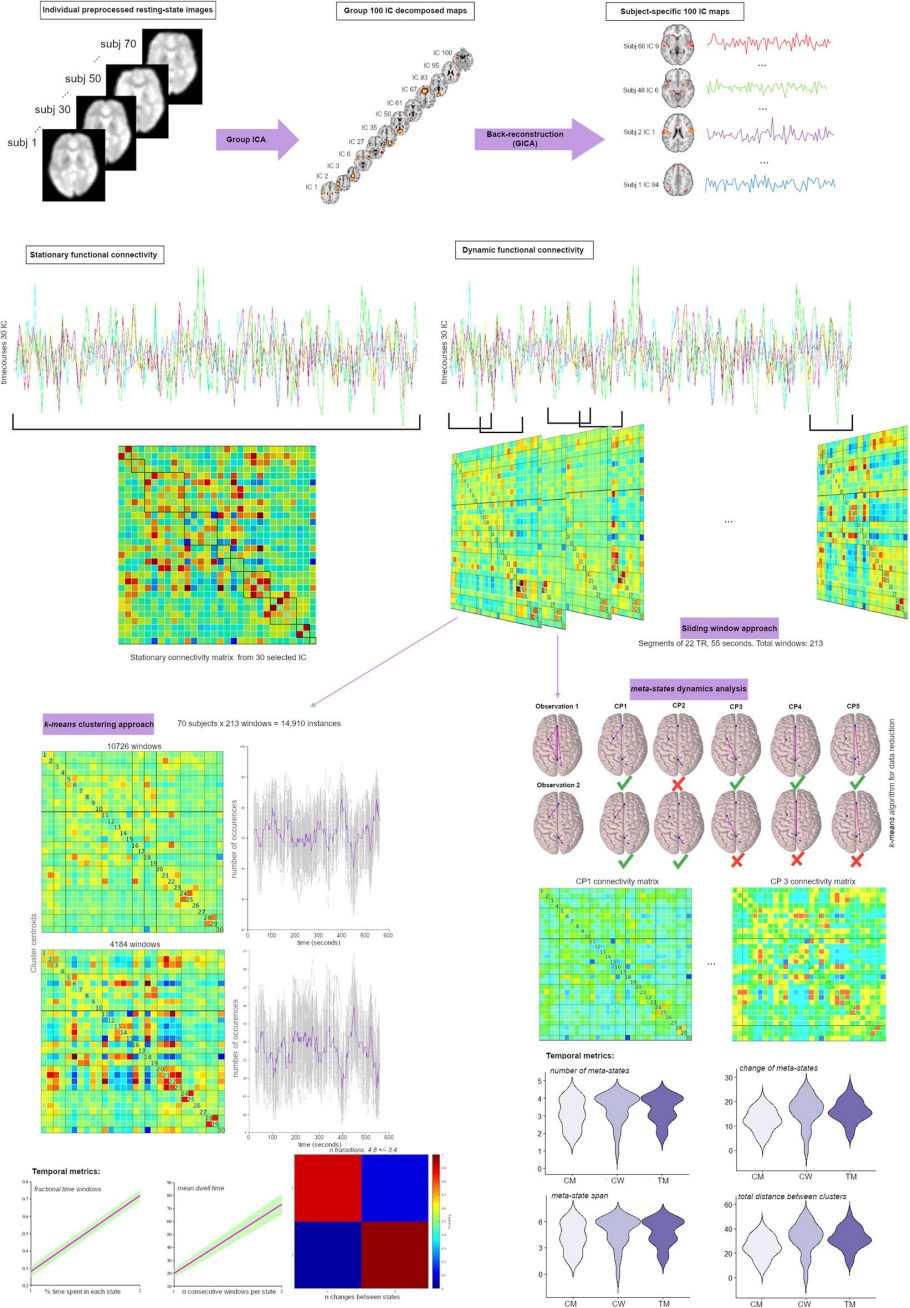
Summary of analyses steps. Group independent components (IC) analysis decomposes resting-state data of the total 70 subjects into 100 IC components. GICA back-reconstruction is used to estimate subject-specific IC maps and time courses. From the stationary functional connectivity (FC) maps, subject-specific time courses were segmented into 55 s (22 TRs) and moved by 1 TR (2.5 s) = 213 windows. These windows were used to calculate the FC covariance matrices using the sliding window approach. Secondly, these matrices (per subject) were used to classify FC into clustered FC patterns (states) using a *k-means* clustering approach. From this technique, we obtained temporal metrics such as fractional time windows, the mean of the dwell time per state and number of transitions. Finally, we applied the meta-state dynamic method that models the windowed FC time courses from the sliding window approach as weighted sums of maximally independent connectivity patterns (CP)^30^. Each CP is recast as a discretized vector of CP weights called meta-state. We used a *k-means* clustering algorithm for data reduction. We obtained the following metrics: number of meta-states, change of meta-states, meta-state span and total distance between clusters (i.e., CP). The Surf Ice software (https://www.nitrc.org/projects/surfice/) was used to plot the brains, and connectivity matrices were extracted from Matlab R2020b (The MathWorks, Inc., Natick, Massachusetts).

FC dynamics analysis was performed^22^ using (1) a sliding window approach to investigate changes in FC across time; then, (2) a *k-means* clustering algorithm was applied to assess the frequency and structure of reoccurring functional connectivity patterns in a *native* space; and finally, (3) we implemented the *meta-state* dynamics method^30^ that factorizes FC dynamics correlations into continuous loading coefficients (Fig. 1).

#### Sliding window approach

We used a window width of 22 (i.e., TRs = 55 s per window) with a Gaussian σ = 3 TRs and slid in steps of 1 TR (2.5 s) resulting in 213 windows. L1 was applied as regularization method^41^ to compute a sparse inverse covariance matrix, and optimized for each subject (1000 repetitions). Finally, FC estimates were Fisher *z-*transformed for each window and they were concatenated resulting in a 30 × 30 × 213 array to continue with the clustering approach.

#### Clustering analysis

To assess the frequency and structure of reoccurring FC patterns (states), we applied a *k-means* clustering analysis using the L1-distance function to the 213 windows per each pair of the 30 IC components (213 × 435 matrix) and iterated 500 times. The resulting centroids were used to initialize a clustering of all data (70 × 213 = 14,910 instances). We explored two different cluster solutions: the 2-states solution according to the silhouette criterion and the 4-states solution according to the elbow criterion and the Dunns index.

Clusters’ reproducibility was established with two non-overlapping split-half samples, and with 5 different sets of randomly selected subjects (Supplementary Fig. 1).

Computed metrics are: *fractional time windows,* as the proportion of time spent in each state; *dwell time,* as the mean time subjects remained in each FC state; and *state transitions*, as the sum of times each state changed from time t−1 to time t, a higher number of transitions represents less stability over time.

#### Meta-state dynamics method

In addition, we report metrics from the *meta-state* dynamics method^30^ as implemented in the GIFT toolbox. This method creates continuous loading coefficients that are discretized using quartile discretization (dimensions are 213 windows × 2/4 clusters × 70 subjects). We chose the *k-means* cluster centroids approach described above to decompose FC data into 2 and 4 connectivity patterns. This method converts the original weighted data to discrete meta-states by replacing each connectivity pattern weight with a value in ± [1,2,3,4] according to its quartile^30^.

Computed metrics are: the *number of meta-states,* as the number of unique windows per subject; *change of meta-states,* as the number of times each subject switched states; *meta-state span,* as the maximum L1-distance between states/clusters; and *total distance,* as the sum of L1-distances between successive clusters.

### Statistical analyses

A general linear model was applied to test the between-subjects effect and Fisher’s Least Significant Differences (LSD) post-hoc multiple comparison testing using IBM SPSS Statistics 26.0.0.1 (2019, Armonk, NY: IBM Corp). In addition, Cohen’s *d* effect sizes were calculated. We also used the threshold-free network-based statistics^42^ to test intergroup differences of the strength of the cluster medians per state. Spearman correlations were computed between the quantitative FC dynamics metrics and the MAIA scores per group.

## Results

### Stationary functional network connectivity

**Figure 2:**
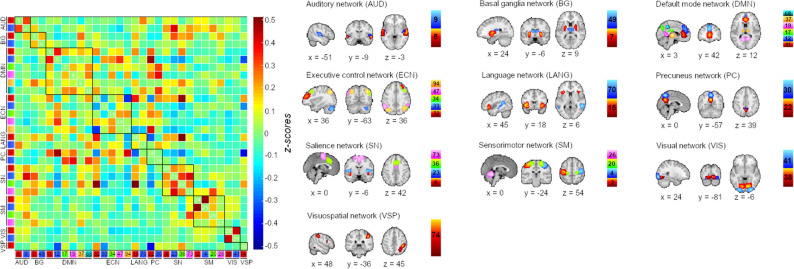
Group IC analysis maps and its functional connectivity. In the left, correlation matrix between the 30 selected IC, black squares in the matrix represent correlations between IC within the same network (intra-network connectivity). In the right, spatial representation of the selected IC divided according to the Findlab atlas^40^. IC maps and matrix were obtained from the GIFT toolbox^22^.

Figure 2 summarizes the 30 IC maps and the stationary FC between them, obtained from group IC analysis and grouped into 10 networks^40^: within *(i) the auditory network,* ICs 8 and 9 located in the superior temporal lobe; with *(ii) the basal ganglia network,* putamen (IC 7) and thalamus (IC 49); within *(iii) the default mode,* precuneus (ICs 17, 19 and 68), paracingulate widening to the superior medial frontal (IC 12), anterior cingulate (IC 11) and thalamus (IC 37); *(iv) the executive control network,* with a right hemisphere frontal-parietal (IC 34) predominance covering the frontal pole (IC 32), lateral occipital (IC 94), angular gyrus (IC 47) and the cerebellum (IC 33); in *(v) the language network,* the right orbitofrontal (IC 15) and the middle temporal (IC 70); *(vi) the precuneus network* (ICs 22 and 30), both with peak coordinates in the right hemisphere; with *(vii) the salience network,* peak coordinates in the insula (ICs 6 and 23), paracingulate (IC 36) and supplementary motor area (IC 73); with *(viii) the sensorimotor network,* the precentral gyrus (ICs 2, 4 and 20) and the cerebellum (IC 26); with *(ix) the visual network*, the occipital (IC 41) and lingual gyri (IC 38); and with *(x)* the *visuospatial network,* the right supramarginal (IC 74). See Supplementary Table 1 and Supplementary Fig. 2 for detailed information of the networks. These 30 IC maps were used to compute FC dynamics’ measures.

### Functional connectivity dynamics results: 2-states solution

**Figure 3:**
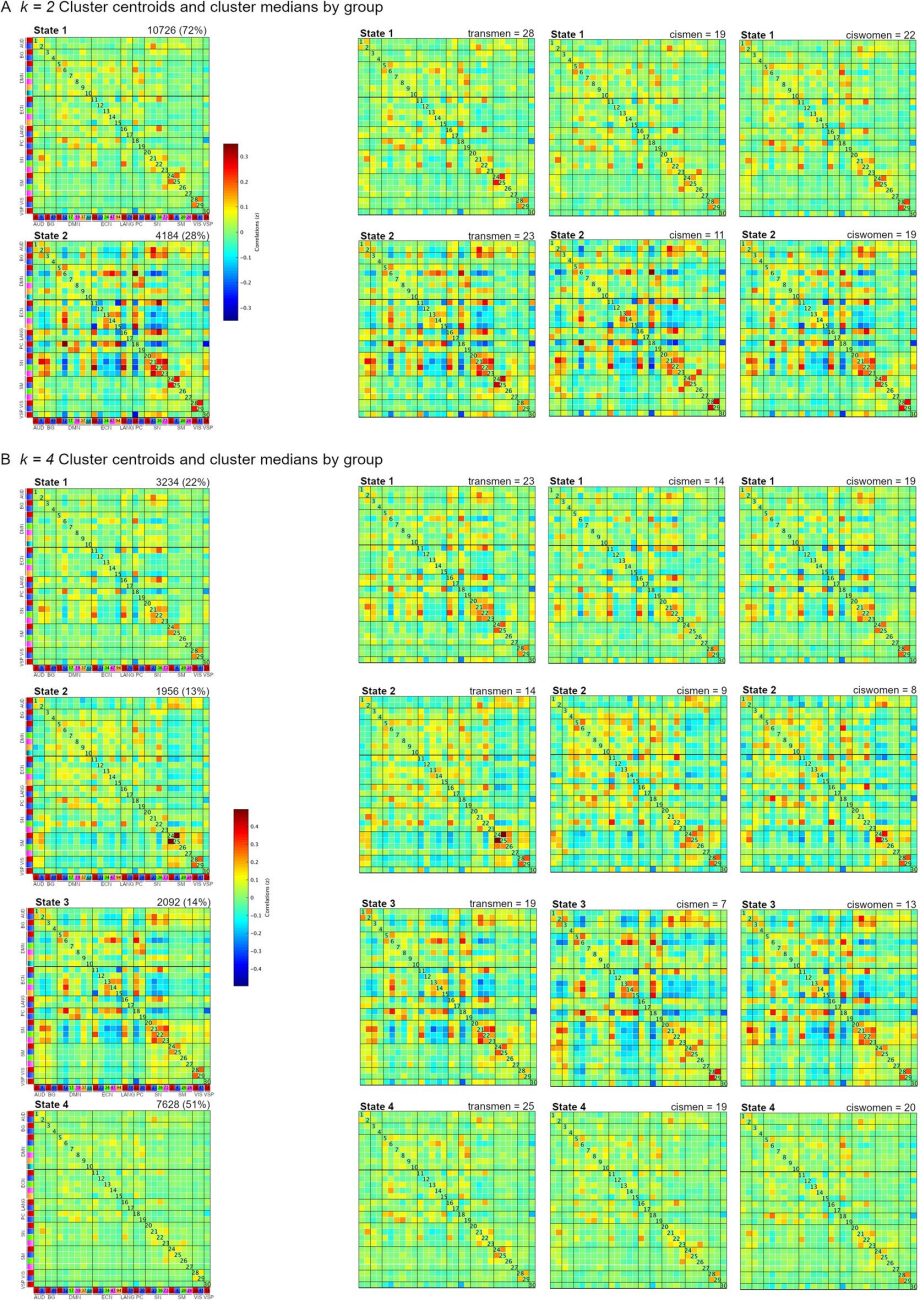
Cluster centroids of the whole sample and group-specific cluster medians. (**A**) Results from the 2-clusters solution (*k* = *2*). (**B**) Results from the 4-clusters solution (*k* = *4*). Matrices were obtained from the GIFT toolbox^22^.

We identified State 1 with a high percentage of occurrences (72%, 10,726 total windows) and relatively sparsely connected; and State 2, less prevalent (28% occurrence, 4,184 windows) but with stronger intra- and inter-network connectivity, and both positive and negative couplings (Fig. 3A leftmost column).

State 1 was characterized by having weaker connectivity than State 2, although there were positive intra-network correlations between nodes of the sensorimotor and the visual networks. The salience network (insula and paracingulate seeds) also presented positive couplings with seeds from other networks, namely, the auditory (insula-superior temporal), executive control (paracingulate-anterior frontal) and the language (paracingulate-orbitofrontal) networks. State 2 presented connected nodes within the executive control, salience, sensorimotor and visual networks while two nodes (frontal-parietal) of the executive control network were anti-correlated. Regarding inter-network connectivity, the salience network had several inter-network correlation strengths with the auditory (insula & supplementary motor area-superior temporal) network, one node of the executive control (paracingulate-anterior frontal) and the language (paracingulate-orbitofrontal) network, and anti-correlations with the default mode (insula & paracingulate-superior medial frontal), executive control (insula-frontoparietal) and precuneus (insula & paracingulate-precuneus) networks. The medial superior frontal component of the default mode positively correlated with frontal-parietal components of the executive control and the precuneus networks, while the node placed in the right supramarginal of the visuospatial network was anti-correlated with the precuneus network.

**Figure 4:**
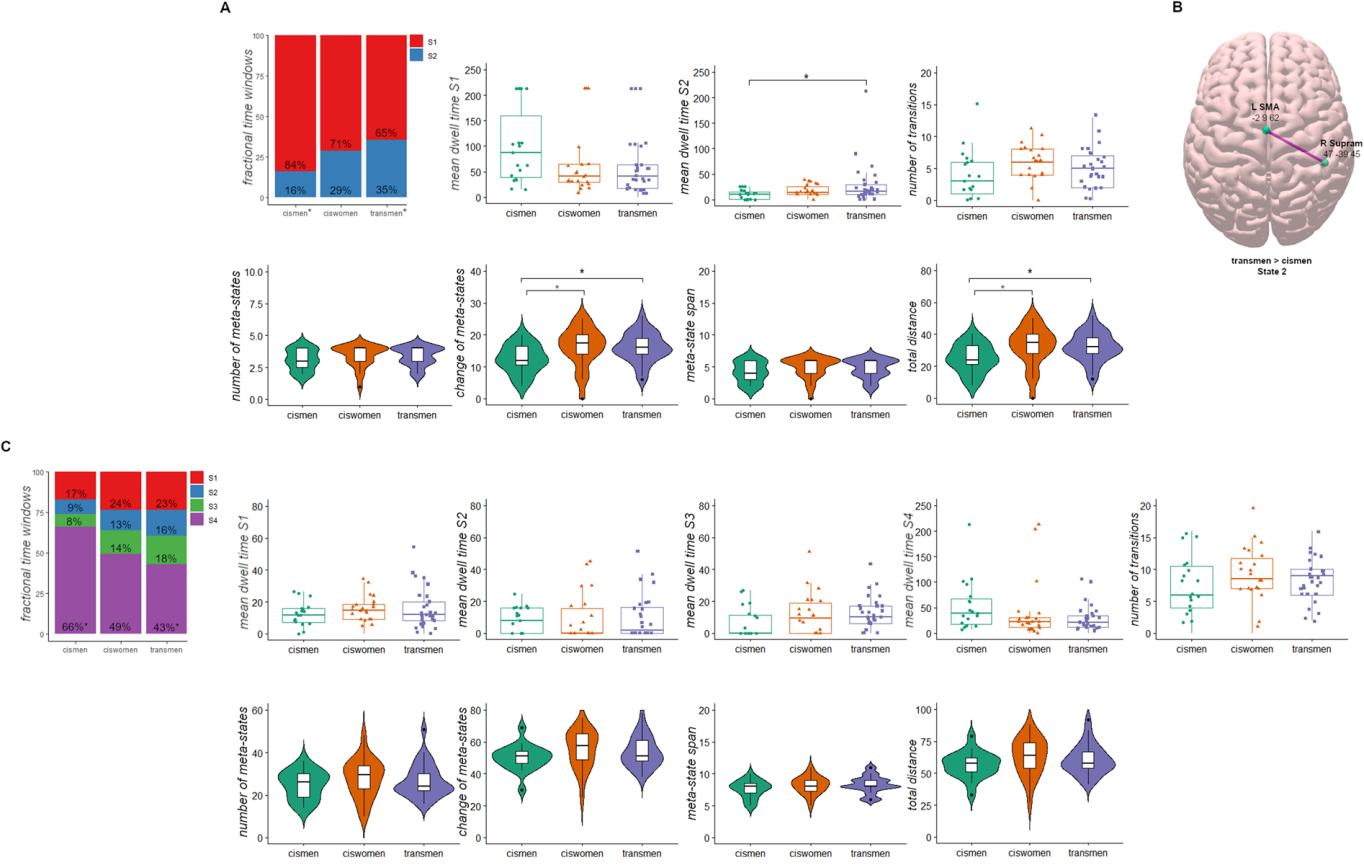
Temporal properties of the FC states analyses. (**A**) Bar plot represents the fractional time windows from the 2-states solution, quantitative temporal metrics from the sliding windows *k-means* clustering approach are represented as box plots, namely dwell time of States 1 and 2 and number of transitions. TM and CM had significant differences in the time they dwelled in State 2. The meta-state dynamic metrics are charted as violin plots. CM differed from CW and TM in the change of meta-states and the total distance between clusters. (**B**) FC differences in State 2 from the 2-states solution between CM < TM using the threshold-free network-based statistics. L SMA, left supplementary motor area; R Supram, right supramarginal. Surf Ice software was used (https://www.nitrc.org/projects/surfice/). (**C**) Bar plot represents the fractional time windows from the 4-states solution, quantitative temporal metrics from the sliding windows *k-means* clustering approach are represented as box plots, namely dwell time of States 1, 2, 3 and 4 and number of transitions. Finally, violin plots represent the 4-states cluster solution of the meta-state dynamics method. Plots were obtained with the ggpubr library (https://cran.r-project.org/web/packages/ggpubr/index.html) using the RStudio v1.4.1106 (PBC, Boston, MA, http://www.rstudio.com/). *Multiple comparison post-hoc test *P* < 0.05.

The Hotelling’s T-squared distribution test did not reach the significance threshold (F = 1.177; *P* = 0.307). The TM group spent a higher proportion of time in State 2 than CM (F = 3.168; *P* = 0.048; post-hoc *P*-value = 0.014; Cohen’s *d* = 0.71). Likewise, the TM group dwelled more time in State 2 than CM (F = 2.651; *P* = 0.078; post-hoc *P*-value = 0.029; *d* = 0.56). CM differentiated from TM and CW in the number of times subjects switched *meta-states* (reduced *change of meta-states,* F = 3.509; *P* = 0.036: CM vs. TM: post-hoc *P*-value = 0.020; *d* = 0.8; CM vs. CW: post-hoc *P*-value = 0.025; *d* = 0.67*),* and reduced distance range between successive clusters, as per the *total distance* metric (F = 3.509; *P* = 0.036: CM vs. TM: post-hoc *P*-value = 0.020; *d* = 0.8; CM vs. CW: post-hoc *P*-value = 0.025; *d* = 0.67, Fig. 4A). There were no significant differences between CW and TM dynamic metrics. All metadata can be found in a repository (10.6084/m9.figshare.14750841.v2).

Finally, we also compared the FC strengths between groups in each state. Within State 2, TM had increased connectivity strength with respect to CM (t = 3.149; *P* = 0.048) between the left supplementary motor area (IC 73) and the right supramarginal gyrus (IC 74, Fig. 4B). No other differences among groups’ cluster medians correlation strengths were found.

### Functional connectivity dynamics analysis: 4-states solution

We identified three states that presented sparse connectivity with focal modularity between specific regions, while one state showed stronger intra- and inter-network positive and negative correlations. State 1 (22% occurrence, 3234 total windows) was characterized by positive intra-network couplings in the salience, sensorimotor and visual networks. As for the inter-network connectivity findings, the salience was also correlated with other nodes’ networks such as the auditory (insula-superior temporal), the executive control (paracingulate-superior frontal) and the language (paracingulate-orbitofrontal) networks similar to State 1 in the 2-states solution. Additionally, the precuneus network’s nodes correlated with the default mode (superior medial frontal) and executive control (superior parietal) networks. Connectivity in State 2 was overall sparse and with a low percentage of occurrences (13%, 1956 total windows) but showed a specific strong positive relation between two nodes (bilateral precentral gyrus) of the sensorimotor network. State 3 also had low frequency of occurrences (14%, 2092 windows) but presented higher strength of couplings. Two salience’s nodes (insula-supplementary motor area) and the visual network (occipital regions) had strong positive correlations, while two executive control’s nodes were anticorrelated (frontal pole-superior parietal). The salience network correlated with one node in the auditory (insula-superior temporal), and one in the executive control (paracingulate-superior frontal); the default mode positively correlated with the executive control (medial superior frontal-superior parietal); and the visuospatial network node anti-correlated with the precuneus network (supramarginal-precuneus). Finally, State 4 was the most frequent (51%, 7628 windows) but displayed the sparsest connectivity across regions (Fig. 3B).

The Hotelling’s T-squared distribution test did not reach the significance threshold (F = 0.701; *P* = 0.841). The proportion of time spent in State 4 differed significantly between CM and TM (F = 3.225; *P* = 0.046; post-hoc *P*-value = 0.014; *d* = 0.74), where CM spent more time in this sparse weakly connected state than the transgender group (Fig. 4C, 10.6084/m9.figshare.14750841.v2).

Finally, there were no intergroup differences in the FC strengths within any state.

### Brain dynamics correlations with interoceptive awareness in transmen

**Figure 5:**
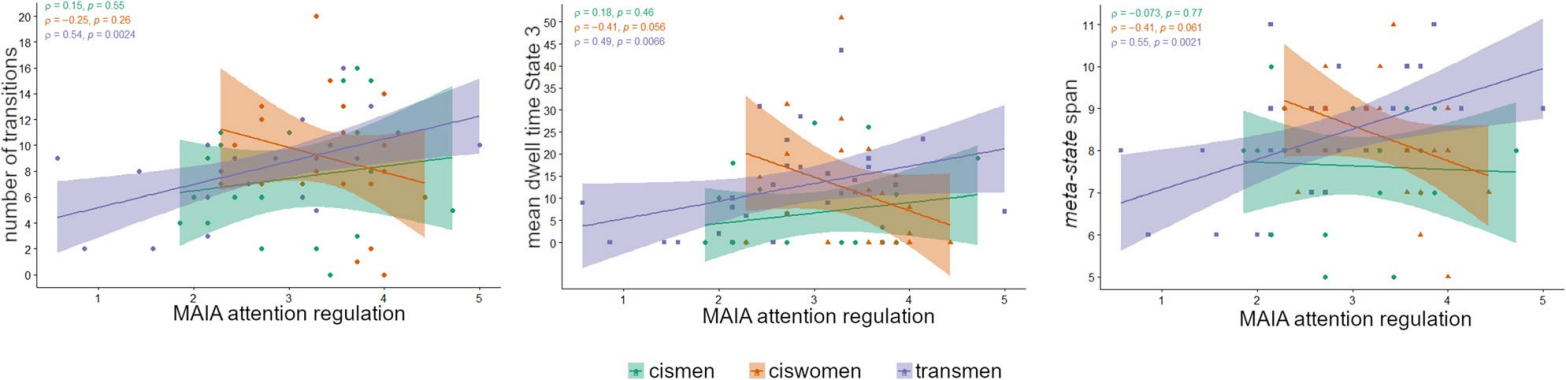
Correlations of the attention regulation Multidimensional Assessment Interoceptive Awareness (MAIA) dimension with metrics of the 4-states cluster solution. Within the transmen group, the number of transitions and the dwell time in State 3 as metrics of the sliding windows *k-means* clustering approach, were positively associated with the attention regulation dimension; there was also a positive correlation between this MAIA dimension and the meta-state span. Correlations are rho Spearman, and shadowed areas represent the 95% confidence interval. Plots were obtained with the ggpubr library (https://cran.r-project.org/web/packages/ggpubr/index.html) using the RStudio v1.4.1106 (PBC, Boston, MA, http://www.rstudio.com/).

We report significant Spearman’s correlations within the TM group between MAIA proxies and brain dynamics metrics. In the 2-states cluster solution, there was no significant association with any MAIA dimension after discarding four outliers. We did find a positive association between the attention regulation dimension (7 items questioning the *ability to sustain and control attention to body sensations* with questions as “I can return awareness to my body if I am distracted” or “When I am in conversation with someone, I can pay attention to my posture”) and the number of transitions, the mean dwell time in State 3; and *meta-state* span in the 4-states solution in the TM group (Fig. 5). These three significant correlations within the transmen group did not survive family-wise error nor the false discovery rate corrections as per the number of MAIA dimensions and FC dynamical measures (10.6084/m9.figshare.14750841.v2).

## Discussion

We identified three overall sparsely connected states and one state with strong positive and negative couplings. Although all groups presented similar connectivity states, TM and CW presented more instability in the temporal *meta-states* transitions than CM; and TM spent more time in the more densely connected state than CM. In addition, TM had stronger connectivity than CM in the coupling between the visuospatial network node and the seed in the supplementary motor area of the salience. TM and CW had a statistically similar profile. Finally, the attention regulation dimension of interoceptive awareness was positively associated with both states and *meta-states* metrics in TM, while such relation was not found in the cisgender groups.

The compelling finding was that TM dwelled more time in the moderately frequent densely connected state than CM, with a medium effect size being the difference between groups smaller than one standard deviation. Remaining significantly more time in this state than in the other sparser state could be related to an increased saliency. The relevant role of the salience network has been previously stressed in terms of explaining gender phenotypes^4^. Moreover, *meta-states* fluidity and range dynamics differentiated CM from the TM and CW. Contrary to recent cisgender works^28^, CM had less *meta-state* dynamism than TM and CW. In our previous stationary FC results^4^ using the same sample^38^, we reported decreased functional connectivity in TM with respect to CM. We could speculate that TM possesses increased integration large-scale properties in expenses of reduced segregation states than CM, resulting in an increased *averaged* connectivity of CM comparing them with TM. However, stationary graph-theoretical results failed to differentiate these two groups.

It is important to note that CW and TM did not differentiate in any FC dynamical measure. Given that there are no prior reports of FC dynamics in transgender people, any speculation should be taken carefully. We could argue that more sensitive dynamical measures are needed to capture the spatiotemporal dynamics underlying gender, and the small number of participants assessed hampered such discrimination. Another explanation could be the presence of sex differences, as transmen were assessed before any gender affirmative hormone treatment, and they had the same assigned sex as ciswomen. Nonetheless, even the more classical battle of sexes in neuroscience is being currently revisited, and the sex and gender concepts are deeply entangled that it may be challenging to resolve such bidirectionality^18,43^. Whichever the explanation, further investigations are needed to elucidate it.

Interestingly, intergroup differences of the State’s 2 FC revealed that TM had increased connectivity between two nodes: the right supramarginal and the left supplementary motor area with respect to CM. We previously reported decreased connectivity of the supramarginal gyrus in TM with respect to CM^4^; and thicker cortex in TM and CW with respect to CM using a different cohort^5^. Functional connectivity differences in the supramarginal gyrus between cisgender^44^ and transgender groups^11^ were also reported in relation to the own body perception^11,44^; and to interpersonal emotion processing^45^. Multimodal translational approaches^46^ are needed for understanding these compelling yet challenging findings contributing to the formation of the *gendered*-self.

We also described one discrete functional configuration with a coupling between the bilateral precentral gyrus of the sensorimotor network. We could associate this *sensorimotor-state* with the self-representation of the typical traits assigned at birth as suggested by the functional hypothesis^10^. Indeed, increased activity in cismen in the precentral cortex in a small cohort of cisgender individuals has been described from a task-based self-body perception task^44^. However, this state was slightly prevalent (13%), and TM’s connectivity did not differentiate from the cisgender groups.

One key element is present between our previous functional work^4^ and the present: the salience network and its interactions. We could describe the salience as a switching network^47^ that interplays mainly but not restricted to the default mode, executive control and itself, as well as the auditory and language networks. Interestingly, when defining the mind-wandering processes, the “general salience network” is associated with an automatic bottom-up salience detection that together with the core of the default exerts automatic constraints on thought; while the executive control exerts deliberate constraints by reducing those automatic constraints controlled by the other two networks^48^.

In line with the frontal predominance described from the *static* node-based connectivity approach^4^, we also observed a frontal involvement between the FC couplings within states. More interestingly, two regions stood out: the insula and the paracingulate. In the transgender literature, projections between the pregenual anterior cingulate cortex and the insula^49^ are related to the own body perception, as well as increased activity in the paracingulate when viewing bodies different from the assigned sex at birth^11^ in TM^10^. The insula is described as a multimodal convergence area implicated in body awareness^50^, and an integral hub^1,47^.

In addition, we found a positive association between the spatiotemporal dynamic metrics and the attention regulation proxy of the interoceptive awareness scale only in the TM group. Interestingly, those TM who were more apt to switch connectivity states and range over greater *meta-states* spaces presented higher interoceptive awareness. We could speculate that the densely connected state (State 3) pertained to a sustained attention regulation to body sensations in relation to the experience of the resting-state acquisition in TM. State’s 3 FC was represented mainly by positive and negative couplings within the *triple-network*^1^. Regionally, mind-wandering processes are associated with the default mode activity, while the change to an awareness of such mind-wandering periods is related to the interplay with the salience^48^. Indeed, it is hypothesized that the salience network “increases the stability of attention over time by constraining the spontaneous movement of attention”^48^. Nonetheless, the relationship between the FC dynamics and attention regulation did not survive any multiple comparison correction, future more directed investigations should be made in this regard.

Among the scarce literature on interoceptive awareness and brain imaging^51–53^, a recent study reported self-regulated sustained attention by modulating the interaction between the salience and the default mode networks^54^. Such studies reinforce the approach that mind, cognition and emotion emerge from the reciprocal interactions of the brain-body signals-external world. In TM such body self-identification requires additional involvement of emotional processing^11,55^.

Among the available hypotheses to explain gender, some try to answer the question of *why* gender is built in the brain and others on *how* the brain works in relation to gender. Within the framework of the *why*, we proposed a neurodevelopmental cortical hypothesis^8^ by means of structural MRI techniques in transgender people before undergoing hormone affirming treatment^5–7^, that was later supported by brain functional MRI data^4^. Based on our previous findings, TM have a mixture of masculine, feminine and defeminized morphological brain traits. Interestingly, cortical thickness of TM differentiated from CM (but not from CW) in a different pattern than that observed when comparing CM and CW. These patterns were also found when using functional data^4^. When shifting the focus on the *how*, a predominant hypothesis suggests that gender incongruence could rest on the functional “*disconnection*” of fronto-parietal regions of the own-body self-referential network, also reporting a cortical thickening of the mesial prefrontal and precuneus cortices^10,33,49^.

In this work, we can observe a similar pattern as we previously described. We previously suggested that sex differences in gender groups would be measure-dependent^8^, conferring each gender a particular phenotype. Regionally, our temporal perspective agrees with the involvement of a *sensorimotor-state* that could be implicated in the so-called own-body perception network^10,44^, and we postulate that the insula and its projections are not only implicated in own-body perception processes in transgender people^49^, but as one of the brain regions that constitutes the core in building a gendered-self.

This novel whole-brain *chronnectome* technique suggests that the underlying brain mechanisms accounting for gender differences are not likely explained by interactions of one sole network, or from a functional “disconnection” of specific brain regions^10,33^. We could hypothesize that the potential FC signatures underlying gender variability rely on a shared dynamical core that orchestrates differently, and in a gender fashion, the functional activity of the rest of the brain. Undoubtedly, disentangling the brain dynamics differences underlying the conceptualization of the gendered-self have a great potential as new dynamical models emerge^56–58^. These models offer a causal mechanistic explanation to the brain dynamics constrained by functional and structural connectivity^56^, that allow to study the information processing across time, space, and scales^57^.

In this work, one important shortcoming is the TR > 2 s, hindering a thorough assessment of the temporal brain dynamics which can be solved by multiband acquisition protocols. Secondly, transgender variants are relatively rare^59^ and therefore, our sample size limited the robustness of the associations with the awareness questionnaire that have no global score. In addition, the lack of differences between CW and TM should be carefully interpreted as our sample size prevents generalization, or more fine-grained functional connectivity dynamics techniques would be necessary for discriminating gender groups. Thus, the present work should be considered exploratory, and it should be replicated in larger samples as the ENIGMA initiative is putting forward with the gender study group^16^. Another limitation would be the non-systematical assessment of sexual orientation for all participants. However, the need to regress out such an effect when investigating gender identity is yet unconclusive^16^. Finally, it is important to highlight that while we attempted to picture the gender differences beyond the cisgender comparisons, we did not include transwomen, genderqueer, or other nonbinary identities. Indeed, political, sociological, and cultural factors were not taken into account, and with this study, we only aimed at finding an association between brain connectivity states and the a priori binary concept of gender. We would also like to stress the need to cooperate and consult with the transgender community when conducting gender studies in order to prevent stigmatization.

In sum, gender group differences presented with a picture where all groups had a similar FC dynamics profile, and CM differentiate from the other two groups, CW and TM which had similar dynamism. We found that FC dynamics underscore the importance of the salience as keystone brain network and its interplay with other networks. In this process, the insula is one relevant region that makes possible the switch between the internal and the external world. We stress the importance of the study of whole-brain network interactions rather than regional connectivity differences of a priori seed-based analysis. Finally, we provide a functional explanation from spatiotemporal evidence that adds up to the neurodevelopmental hypothesis proposing different brain phenotypes, mainly differentiating CM from TM and CW.

## Supplementary Information

Supplementary Information.
