# Supplementary material for: Brain connectivity dynamics in cisgender and transmen people with gender incongruence before gender affirmative hormone treatment
Source: Sci Rep. 2021 Oct 26;11:21036. doi: 10.1038/s41598-021-00508-y (PMC8548343; doi:10.1038/s41598-021-00508-y)
Supplement: Supplementary file 1 — Supplementary Information. [file 41598_2021_508_MOESM1_ESM.pdf]

Reproducibility of the clustering approach

Supplementary Figure 1 Reproducibility of the estimated dynamic FNC states of the 2- and 4-states solutions

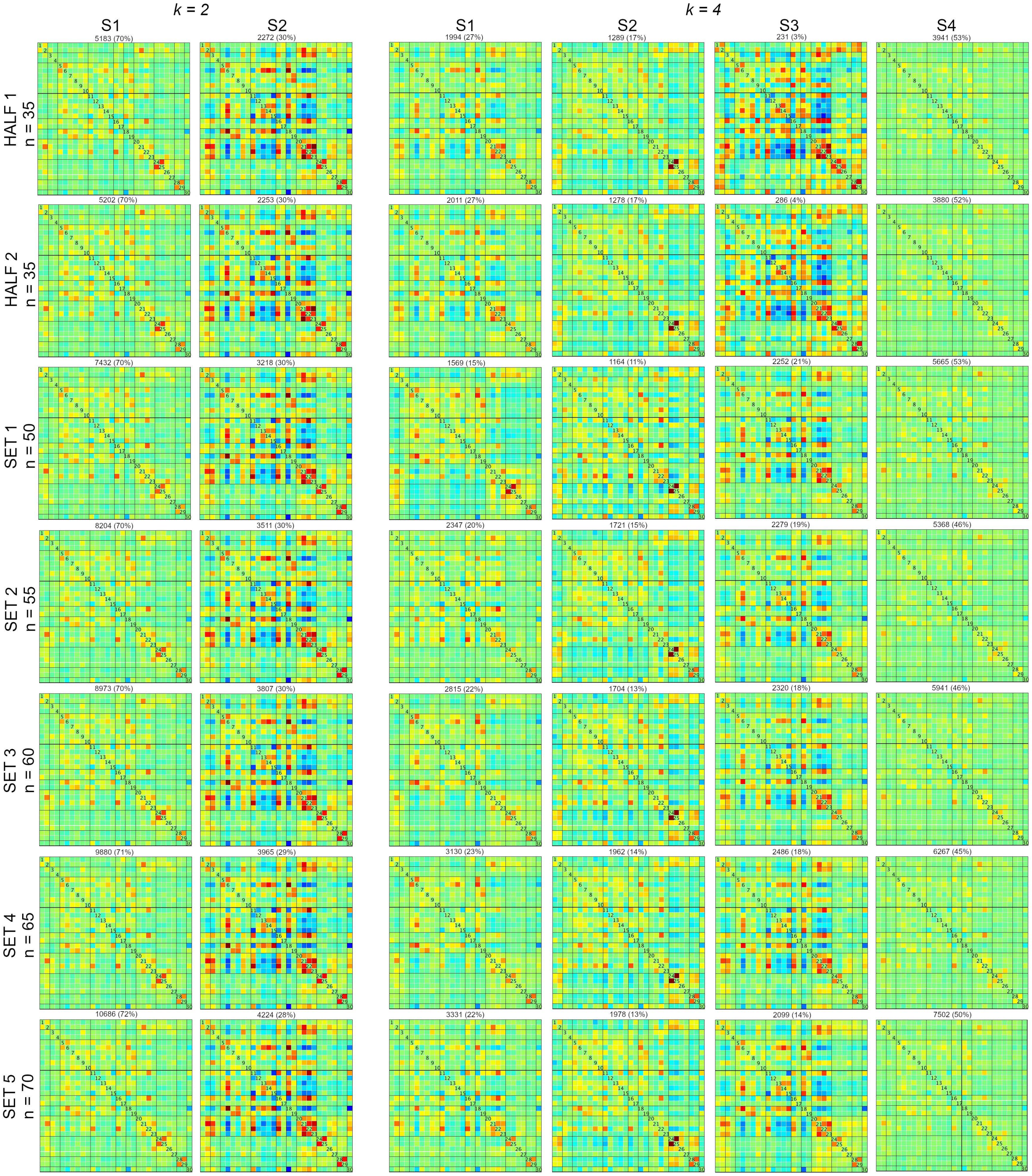

Cluster centroids for split half and random resamples for the 2-states cluster solution (k = 2) and the 4-states solution (k = 4). Subjects were randomly resampled in 5 sets of 50, 55, 60, 65 and 70 and 2 more sets of 35 subjects each (split-half samples). *K-means* clustering algorithm was applied as described in the methods section for the whole sample.

Independent component maps from group-ICA

Supplementary Figure 2 Independent component maps

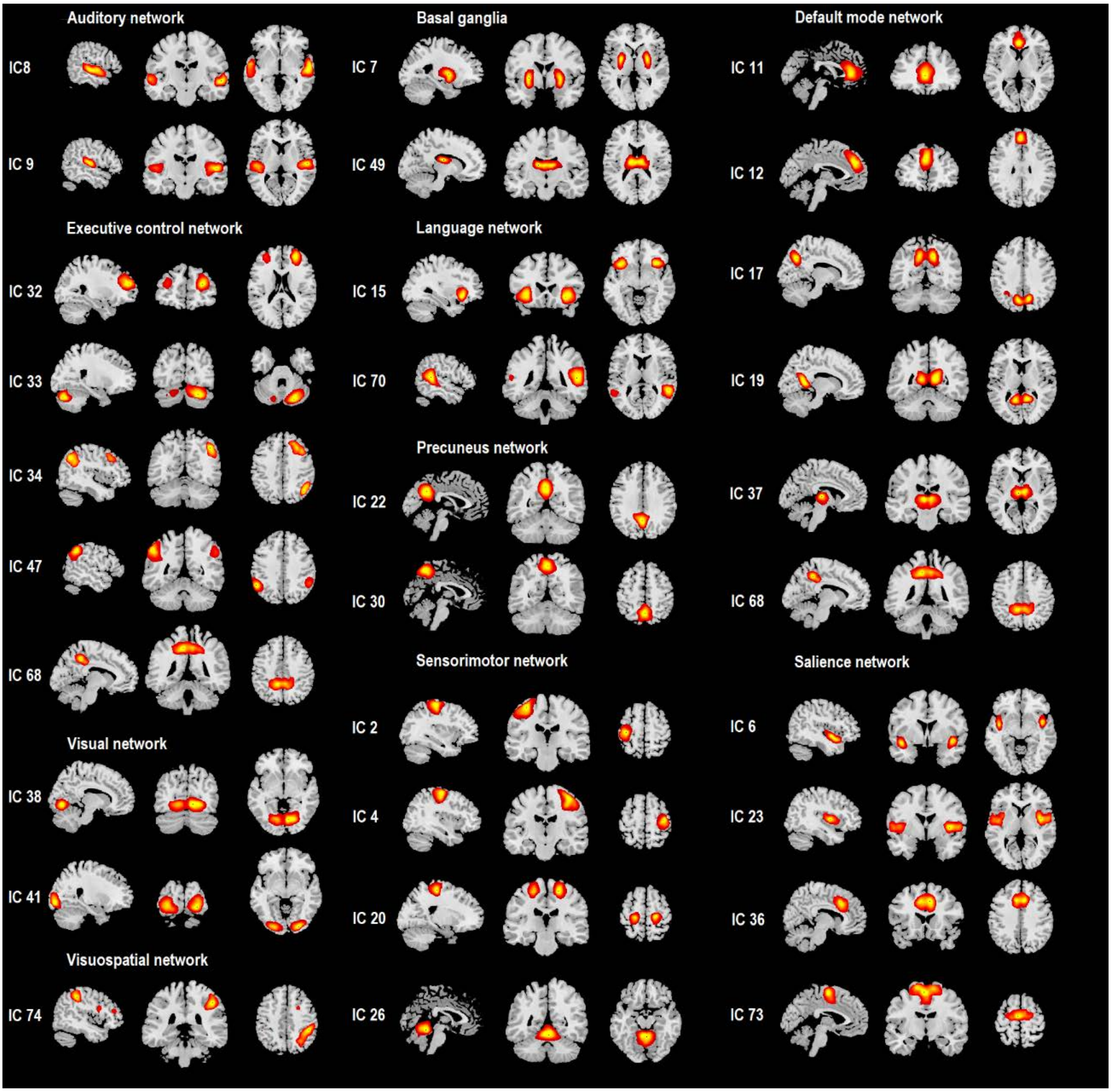

Independent components (IC) maps grouped into 10 networks as in the Findlab atlas (Shirer et al., 2012). Maps were thresholded at t = 4.

Supplementary Table 1 Peak coordinates of the independent components extracted from ICA

Network	t-max stat	Brodmann/cerebellar area	MNI coordinates (x y z)
**Auditory network (AUD)**			
IC 8 right superior temporal	14.383	22	59 -18 -5
IC 9 right planum temporale	16.884	41	54 -17 5
**Basal ganglia network (BG)**			
IC 7 left putamen	20.685	-	-26 0 0
IC 49 left thalamus	18.406	-	-12 -14 15
**Default mode network (DMN)**			
IC 11 right anterior cingulate	22.272	24	0 38 3
IC 12 left paracingulate	20.785	9	-2 47 29
IC 17 right precuneus	27.295	7	12 -65 36
IC 19 right precuneus	25.253	23	12 -51 8
IC 37 left thalamus	18.400	-	-5 -20 3
IC 68 left precuneus	14.670	31	-8 -44 44
**Executive control network (ECN)**			
IC 32 right frontal pole	16.641	10	29 50 18
IC 33 right cerebellum	19.070	Crus I	24 -80 -33
IC 34 right angular gyrus	18.134	39	44 -63 41
IC 47 left angular	19.344	39	-53 -51 39
IC 94 right lateral occipital	12.544	39	38 -62 42
**Language network (LANG)**			
IC 15 right orbitofrontal	16.682	47	33 23 -9
IC 70 right middle temporal	18.945	22	54 -44 9
**Precuneus network (PC)**			
IC 22 right precuneus	23.355	31	0 -56 32
IC 30 right precuneus	18.602	7	2 -59 53
**Salience network (SN)**			
IC 6 right insula	18.209	13	45 2 -9
IC 23 right insula	13.916	13	41 0 2
IC 36 right paracingulate	13.073	8	8 20 38
IC 73 left supplementary motor area	22.151	6	-2 -9 62
**Sensorimotor network (SM)**			
IC 2 left precentral	18.730	4	-35 -23 59
IC 4 right precentral	20.844	6	39 -17 59
IC 20 right precentral	19.336	4	21 -24 60
IC 26 right cerebellum	22.287	Vermis IX	2 -51 -17
**Visual network (VIS)**			
IC 38 right lingual	18.849	18	11 -75 -6
IC 41 right occipital	18.778	18	26 -92 -8
**Visuospatial network (VSP)**			
IC 74 right supramarginal	17.612	40	47 -36 45

IC, independent component.
